# Animal Sentience: Where are We and Where are We Heading?

**DOI:** 10.3390/ani2040628

**Published:** 2012-11-14

**Authors:** Helen Proctor

**Affiliations:** World Society for the Protection of Animals, 222 Grays Inn Road, London WC1X 8HB, UK; E-Mail: helenproctor@wspa-international.org

**Keywords:** advocacy, animal welfare, anthropomorphism, cognition, consciousness, sentience

## Abstract

**Simple Summary:**

Animal sentience refers to the ability of animals to experience pleasurable states such as joy, and aversive states such as pain and fear (Broom, D.M. *Dis. Aquat. Org. ***2007**, *75*, 99–108). The science of animal sentience underpins the entire animal welfare movement. Demonstrating objectively what animals are capable of is key to achieving a positive change in attitudes and actions towards animals, and a real, sustainable difference for animal welfare. This paper briefly summarises understanding of animal sentience through the ages. There follows a review of the current state of animal sentience, and concluding thoughts on its future in regards to animal welfare.

**Abstract:**

The science of animal sentience underpins the entire animal welfare movement. Demonstrating objectively what animals are capable of is key to achieving a positive change in attitudes and actions towards animals, and a real, sustainable difference for animal welfare. This paper briefly summarises understanding and acceptance of animal sentience through the ages. Although not an exhaustive history, it highlights some of the leading figures whose opinions and work have most affected perspectives of animal sentience. There follows a review of the current state of animal sentience, what is known, and what the main limitations have been for the development of the study of sentience. The paper concludes with some thoughts for the future of the science, and where it should be going in order to most benefit animal welfare.

## 1. A Brief History of Animal Sentience

Discussions over whether animals are conscious beings, capable of feelings such as pain, pleasure and suffering, have been recorded as far back as records allow. For example, ancient thinkers, Plutarch, Hippocrates and Pythagoras were all advocates for the fair treatment of animals. Their urgings were based on their understanding of the capacity of animals to feel pain and suffer [1]. During the renaissance period (*ca*. 14th–17th century), a number of perspectives were proposed on the topic. These included the infamous view from Descartes, who saw animals as automata, incapable of feeling or suffering [2]. Descartes' way of thinking was soon overshadowed by the drive for intellect and reason that was characteristic of the 18th century and the age of the Enlightenment. This period saw great changes in how animals were viewed, with a number of philosophers discussing the ability of animals to suffer [3]. For example, Jeremy Bentham famously wrote in 1789, “The question is not, Can they reason? nor Can they talk? but Can they suffer?” [4]. British politician James Burgh also wrote about the capacity for animals to suffer, and was particularly concerned with the impact that a lack of knowledge may have on children. In his book, “Dignity of Human Nature” [5], Burgh wrote; “Children ought to be convinced of what they are not generally aware of, that an animal can feel, though it cannot complain, and that cruelty to a beast or insect, is as much cruelty, and as truly wicked, as when exercised upon our own species.” This compassionate and reasoned understanding of the experiences of animals continued in to the 19th Century, a period which was primarily characterised by Darwin. Darwin often spoke about the capacity of animals to feel pain, and their many similarities to the human animal. He accepted without question that animals were capable of many emotions and experiences, both similar and different to humans. Darwin even proposed that at least some animals were capable of self-consciousness [6]; a trait once generally assumed to be solely human. Indicators of self-consciousness, such as mirror self-recognition, have since been demonstrated in great apes, dolphins, elephants and magpies [7,8,9,10,11].

The early to mid-20th Century was characterised by the behaviourist movement, a discipline that influenced perceptions of animals for around 70 years, and even today has a lasting impact. Watson, who founded the Behaviourist School of Psychology in 1913, was driven by the idea that only observable behaviour should be studied, discrediting any subjective experiences, intention, or emotions in animals [2]. Contesters of the behaviourist theory at that time included McDougall, who argued that emotions were what drives behaviour, not inbuilt reflexes [12]. Following this time, there were a number of developments that highlighted the importance of sentience. In the 1960’s, the book “Animal Machines” was written. In her book, Ruth Harrison exposed the realities of intensive farming at the time, and the suffering of the animals within them [13]. In response to this, the UK Government set up the Brambell Committee in 1965, which looked specifically at the welfare of animals in farming systems. The committee understood the importance of sentience, and ensured that all assessments took in to account both the feelings and behaviour of the animals [2]. Since then, there has been a notable increase in the number of publications concerned with animal welfare and the recognition of sentience. However, despite this long history of thinking about animals as conscious beings, the science of animal sentience is still a burgeoning topic. What is known today is still limited, for reasons discussed in the following sections. 

## 2. Difficulty of Measuring/Proving Sentience

One of the key issues with understanding sentience and demonstrating its existence at a scientific level, is that the concept relates to a being’s own thoughts, feelings and emotions, none of which can be fully understood or described by physiological processes or anatomical structures. Neuroscience can tell us, for some animals, which parts of the brain produce emotions, and we can make educated inferences about which physiological indicators are evidence for the feelings and experiences associated with sentience. The problem is, however, we cannot know exactly what, or how another is feeling [14]. This applies to both humans and animals, and means that it can be difficult to ultimately prove the capacity for sentience. This is particularly difficult for animals as they lack the power of speech to convey their feelings. As a result, sentience is often described as anthropomorphic assumptions, and its credibility as a science has suffered. This has had negative impacts on the development of the science and our understanding of sentience. Scientists in the field are often hindered by this, and continue to seek unquestionable proof of sentience in animals. However, because sentience is characterised by personal phenomena, and it cannot be known with absolute certainty what another is feeling, it does not lend itself to this type of rigorous analysis. This is often seen as an inherent flaw in the science of sentience, and one which risks the credibility of any conclusions drawn. Yet, sentience is not actually alone in encountering this drawback. Human psychology may also suffer from the inability to know another’s subjective thoughts, despite the seemingly advantageous shared language. For instance, humans are subject to false reporting of their own emotions, whether intentional or not. Furthermore, the field of psychology is often reliant on making assumptions regarding the mental state or thought processes in another human being. In fact, according to Professor Marc Bekoff, within science, there are very few subjects that we know everything about, all of the time [15]. This means that the scientific study of sentience is no different from the rest of science. Despite these difficulties, researchers should continue to strive for robust and valid evidence of animal sentience, and not allow the lack of a shared language to constrain the interpretation and application of the evidence. 

## 3. Anthropomorphism

Another of the key limitations to the acceptance and development of the science of animal sentience is the fear of anthropomorphism; the attribution of human characteristics to an animal. The concern over anthropomorphism really began following the behaviourist movement, when there was a drive to think of animals only in terms of behaviour and to not attribute any subjective feelings or experiences to them [16]. Fortunately, science has moved on since then, but the fear of being anthropomorphic still remains. Some avoidance of anthropomorphism is necessary, as misuse can undermine the science of sentience, however, complete avoidance of anthropomorphism can also be unhelpful, and in many ways impossible. Our anthropomorphic tendencies may even be an innate part of our hereditary make-up [16]. Kennedy suggests that the ability to predict and control the behaviour of other animals may have been an advantage selected for in natural selection [16]. Evidence of our anthropomorphic tendencies is apparent throughout our dealings with, and perceptions of animals. Just as we assume we know what another human is feeling, we often make the same assumptions for non-human animals. For example, an owner may say about his or her pet dog that; “He is sad because we left him at home all day”. Anthropomorphism is also largely featured in our childhoods, as we are bombarded with animals in cartoons who dress and talk like humans. Furthermore, anthropomorphism is often used to engage both children and adults with animal welfare and conservation issues. The need for us to relate to animals in this way is also apparent in our interactions with companion animals. For instance, in many cultures, dogs and cats and other non-human animals are viewed as family members, providing a great source of companionship, and many are even dressed up in specially designed outfits. Anthropomorphism appears to be unavoidable, because not only is it a part of us culturally, hereditarily or both, it is also apparent and often necessary in how humans make sense of and relate to animals [16]. 

Science can never be entirely free from anthropomorphism, nor should it be. Complete abstinence from anthropomorphism would hinder scientific curiosity and exploration. It is the thinking about animals through our own experiences that gives rise to many of the research questions regarding their capabilities. Absolute avoidance would also mean that any traits found in both human and non-human animals would have to be labelled differently, in order to differentiate between them. This can and is already being done within science, and the result is a decrease in the meaning and value of these discoveries of animal sentience. It also seems illogical to do this when there is evidence to suggest that these emotions or traits are fundamentally the same in both humans and the non-human animals in question [17]. There is also a greater price to pay for approaching sentience in this way, and that is the loss of the relevance to humans and human actions. The recognition of non-human animal emotions and the naming of them with the same labels as human emotions, paints a far more vivid picture and argument for compassion than a sterile, non-meaningful term does. This is particularly important given that the science of animal sentience has a more important role to play than just scientific discovery. There is an ethical motivation behind understanding what animals are capable of, and this should be a key consideration. Anthropomorphism is unavoidable within animal sentience science. It is a fundamental part of our interactions and perceptions of animals and a part of human nature. Therefore, rather than avoid it, anthropomorphism should be used responsibly and effectively, to add meaning to the science of animal sentience. Improving our scientific understanding of animal sentience is essential if we are to make lasting, sustainable improvements to the treatment of animals. The science of animal sentience must strike the balance between science and ethics. This needs to be done without compromising scientific integrity, but still ensuring the best outcome for animal welfare. 

## 4. Sentience and Cognition

The attribution of sentience to animals can also be hindered by the common misconception that the capacity for sentience is linked in some way to a species’ cognitive ability. Cognition refers to the mental action or processes by which animals perceive, process and store information [14]. Sentience, on the other hand, refers to the capacity of an animal to have feelings, and to be aware of a variety of states and sensations such as pleasure and suffering [18]. It is often assumed that cognition and sentience are inextricably linked, in that cognition automatically implies sentience. Indeed, evidence of higher cognitive abilities such as theory of mind and language, have previously been used as a basis for advocating for the rights of certain species such as the great apes [19]. Cognition is not actually a prerequisite for sentience, and it can be demonstrated independently [20]. For example, a computer and a non-human animal may both be able to perform the same complex task without any cognitive processes taking place [20]. 

Brain size, and the presence and size of a cerebral cortex have often thought to have been correlated with sentience. In fact, some have even argued that the perception of pain is impossible without the cerebral cortex [21]. Increasingly, studies have demonstrated that this is not the case, and that non-mammalian animals without a cerebral cortex can feel emotions and pain, and possess complex cognitive abilities [18,22]. Even within mammals, neurological evidence suggests that at the very least the basic emotions are not reliant on a large cortex. Instead, the evidence suggests that emotions are generated from the sub-corticol internal brain regions, which are found to be similar across species [23]. Total brain size has also been shown to be a poor indicator for both intelligence and sentience [18,22,23], and many now argue that it should be the complexity of the brain’s function that is considered in regards to welfare, rather than its size [18,22,24]. 

Defining sentience through cognitive ability, however, can potentially be harmful to animal welfare. If species who are deemed cognitively advanced are automatically credited with the capacity for sentience, what does that mean for those who aren’t [25]? Would their capacity to suffer be discredited completely? Where should the line be drawn, and with what criteria? Given that the evidence shows cognition to not necessarily be an accurate indicator of sentience, approaching animal welfare in this way could risk sentient species being disregarded due to their lower cognitive ability, rather than their capacity to suffer. Instead of attempting to define sentience through cognition, a wiser approach would be to utilise the knowledge and understanding of animal cognition to reduce suffering, and to increase the positive states of animals who are known to be sentient [14,25]. For example, using knowledge of cognitive processes to understand whether an animal can remember a positive or negative experience, and to predict how he or she will react to similar experiences in the future, can be used to positively impact their future welfare [2,26]. An understanding of cognition can therefore be helpful and beneficial in improving welfare, but it should not be used as a sole measure upon which protection is offered or denied.

## 5. Where are We Now?

### 5.1. Vertebrates

Our knowledge is still limited when it comes to understanding the complexities of sentience and its presence and form across the taxa. Currently, most is known about the vertebrate species, as much of the research to date has focused on them. Today it is generally accepted that at least the vertebrate species are sentient [18,23,24,27]. This is supported by the existence of animal protection legislation around the world, as many national animal protection laws seek protection for all vertebrates and even some invertebrates [27]. This is primarily due to the universal presence of a central nervous system and the similarity of the neurons and brain structure across the taxa [23]. In addition, scientists are now finding complex neurons, which were once believed to be unique to humans, in several species of cetaceans, primates and elephants [28,29,30]. One exclusion to this rule however, appears to be the fish. Despite the fact that fish are often protected by legislation, there still remains to be some debate over their sentience [31]. Some scientists have argued that fish are incapable of suffering and feeling pain because of the marked difference of their brain structure to human’s [21]. This argument, however, is not supported by the current literature, which comprises a growing number of studies that have looked at both nociception and pain in fish [32,33,34]. For example, in one study, scientists found that when a painful solution of bee venom or vinegar, was applied to the mouths of rainbow trout, they behaved in a way that was indicative of pain. The study found that the trout were less likely to be fearful of a novel object that was added to the tank, compared to the control subjects. These results indicated that their attention levels were impacted by their experience of pain. Furthermore, they found that these behaviours stopped and the trout became fearful again when the analgesic, morphine was administered [32]. In their review, Braithwaite et al found that existing research on fish showed that not only are fish capable of nociception, but that they meet all of the criteria thought necessary for experiencing pain in a meaningful way [35]. The authors concluded that although their experience of pain may not be the same as human’s, it is still meaningful to them, and it is therefore important to protect their welfare [35]. The idea that fish would be incapable of suffering, due to their lack of a cerebral cortex, also holds little strength when looked at from an evolutionary perspective. Feeling pain, as opposed to just nociception, would be a selective advantage for animals, as it would help to facilitate meaningful learning and thought processes beneficial for survival. It would also be limiting to think that they could not develop such capacities from other anatomical structures, just as many species have developed senses very different from humans both with and without sharing a similar central nervous system [24]. 

There have been numerous studies looking at the experiences of animals, and as a result there is a good understanding of what animals, or at least the vertebrates, are capable of experiencing. Understanding how animals can suffer, and what emotions they experience, is instrumental for improving their welfare and the legislation and practices affecting them. In addition to this, more is being discovered about the remarkable abilities of different species, and scientists are learning just how many commonalities there are between us. For example, research has shown that chimpanzees can be generous [36], that mice, rats and chickens demonstrate empathy [37,38,39], several species show optimism and pessimism [40] (starlings), [41] (dogs), [42] (honeybees), and that sentient animals experience pleasure and happiness [43]. Understanding the true spectrum of abilities and experiences of animals is not only fascinating from a scientific point of view, but it is also crucial in making necessary advancements in animal welfare. Historically, sentience research has been primarily mammal-centric, and what is known about reptiles, fish, the majority of bird species and most of the invertebrates is still very limited. This is largely due to the inherent difficulties associated with measuring stress and emotions in these taxa. Nevertheless, considering the vast numbers of these animals that are traded, farmed, slaughtered and bred, it is imperative that further work should be performed in this area. 

To date, the majority of studies on animal sentience have focused on the more negative aspects of experience, such as pain and suffering. This research has provided valuable evidence and impetus to make positive changes in practice, but to truly improve animal welfare it is important to understand and address a whole spectrum of needs. Given that sentient animals are thinking and feeling beings, their needs and desires will change constantly. It is therefore not possible to always correctly assume what an animal would prioritise at any one point, as a decision may depend on any unknown factor. Studies such as Harlow’s infamous experiments with infant rhesus macaques [44], and more modern preference tests [45], have clearly shown us that our assumptions of what an animal would prioritise or choose in any given situation can often be wrong. Legislation often ensures that the basic needs of animals, such as food, shelter and medical care are considered, but when it comes to the psychological needs of animals this is often a last thought. There is a strong need to fully understand how animals are motivated, and what they are capable of understanding and feeling, so that their welfare can be improved beyond the provision for their basic needs. 

### 5.2. Invertebrates

Invertebrates are treated very differently from their vertebrate counterparts, and are generally assumed incapable of experiencing pain [46]. Any behaviours appearing to dispute this assumption are often dismissed as automatic responses to stimuli, rather than conscious feelings [47]. There has been very little research to support or contest this assumption, yet the belief remains to be strongly held [47]. The line between invertebrates and vertebrates was initially drawn due to the differences in their anatomy. The invertebrates lack the particular physical characteristics often thought to be responsible or essential for sentience, such as the central nervous system and certain brain structures [23,24,47]. More than just a general perception, these assumptions have led to legislation within many countries excluding invertebrates from their sphere of concern [48]. As a result, invertebrates are treated in ways which would be deemed as cruel and inhumane if they were involving vertebrates. Fortunately, research on invertebrates is increasing, and it is becoming apparent that at least some of the invertebrate species are indeed sentient. In his review paper of invertebrate research, Sherwin argues that findings from invertebrate studies are often interpreted differently to those from vertebrate studies [47]. Sherwin goes on to suggest that if the rules of argument by analogy were applied to these findings, in the same way they are to vertebrate studies, then many of them would provide strong evidence for invertebrate sentience [47]. This would have enormous implications for how invertebrates are treated, and it would mean that both legislation and general attitudes towards invertebrates would need to shift in line with this new understanding and ethical concern. 

One case which emphasises the need for further investigation is the cephalopods. In the last decade or so, research has demonstrated that these animals, once thought to be incapable of experiencing pain, are actually highly intelligent, sentient beings, capable of suffering and many other complex emotions [49]. This has led to the inclusion of cephalopods in some countries national legislation. For example, in 2013, the UK’s Animals (Scientific Procedures) Act (1986) will be amended to extend the protection from the common octopus, which was added in 1993, to all live cephalopods used in experimental procedures. Understanding whether or not these animals can feel pain and suffer is of utmost importance to their welfare, especially when considering that cephalopods are used extensively in research and for food. There is however, much more to know about these species in order to ascertain what constitutes good welfare for them. 

The welfare of crustaceans, or more specifically Decapoda, has also received a great deal of interest in recent years, with a number of studies looking at their ability to feel pain. In their review of these studies, Elwood et al. claim that if we were to use argument by analogy, like we often do for vertebrates, the evidence would lead to the conclusion that Decapods can indeed feel pain and suffer [24]. Like many other animals, crustaceans lack a cerebral cortex, and as a result of this, some have argued that they must be incapable of feeling pain [21]. Elwood and others contest this proposal, arguing that the same function can arise in different taxa using different morphology [24,32,35]. Elwood et al. uses the example of crustaceans’ visual systems to illustrate their point. Crustaceans have excellent vision, despite the marked difference between their nervous system and that of the vertebrates. They argue that it would be illogical to assume that crustaceans lack the ability to feel pain, just because their systems differ from ours. In fact, crustaceans demonstrate in a number of ways that they can feel pain [24]. For example they learn to avoid painful stimuli [50] (crabs), [51] (crayfish), perform behaviours indicative of experiencing pain, such as rubbing [52] (glass prawn) and autotomy [24], and respond to analgesics in a similar way to vertebrates [52]. This is certainly an important area that requires further research and attention, particularly considering the numbers of crustaceans used for food and research.

Establishing whether or not invertebrates can feel pain and suffer is important to ensure their well-being. It is also important to understand what emotions and sensation they are capable of experiencing, and what is important to them. It may be impossible to know exactly what another being experiences or how it feels to them, but that should not stop research aimed at understanding what they are capable of, as this is fundamental to improving their welfare. What is clear is that we don’t have all of the answers yet, and although it may be unwise to assume sentience in all animals without strong evidence, there is certainly a need to be open to what evidence we do have, to act accordingly and to concentrate on filling the gaps. Invertebrates comprise 99% of all animals and billions are used every year for food and research, and many are classed as pests [49]. Attention on invertebrates is increasing as the above examples demonstrate, but nevertheless it is important that research in this field continues on this upward trajectory. We have an ethical obligation to know whether or not the invertebrates we eat, experiment upon and kill are capable of suffering, and if so, then we need to know what constitutes good welfare for them. 

## 6. Where do We Go from Here?

The focus on animal sentience within the scientific community has been steadily increasing over the past few decades. With this increasing trend it is important to look at where it should be heading to most benefit animal welfare. 

### 6.1. Humane Research in to Sentience

As the scientific knowledge on sentience continues to grow, and we understand more and more about the impact humans have on animals [53], it becomes increasingly unethical and illogical to continue to cause animals harm. One issue in animal sentience science is the need to impart suffering on another being in order to demonstrate whether or not he or she can suffer. This research has of course had an important part to play, it has led to significant changes both in legislation and practice, affecting how we treat and use animals in various industries. Nevertheless, it does seem to be a moral paradox in that by continuing to seek this information we may be causing pain and suffering to animals in a bid to prove their sentience. What if there was another way? If the focus was to be on the other, more positive aspects of sentience, such as their ability to feel joy, then this would not only be beneficial in terms of advancing our knowledge in a relatively unexplored area, but it would also lend itself to humane research. Admittedly this is not an easy feat, but it is certainly one which deserves further attention and exploration. Scientists are by nature, extremely creative people, and they may, should they wish to, find systematic and reliable ways in which to study animals in this manner. Furthermore, if we were to use subjects who are already in captivity, such as farm animals, companion animals and sanctuary animals, this could provide us with the necessary research opportunities, without needing to breed animals specifically for research. Anecdotal evidence from studying animals in the wild can also be a valuable starting point for non-invasive research in to sentience. If given the opportunity these anecdotes can then be explored further, through robust methodology, and turned in to valuable, insightful data. These types of observations shouldn't be ignored as these are often the ones which provide the researchers with a deeper, richer knowledge of their subjects, and a better understanding of their emotional capabilities. 

### 6.2. Moving on from the Mammal-Centric Approach

It is also time to move away from the mammal-centric focus of previous research, and to identify non-invasive ways of demonstrating sentience in birds, reptiles, fish and invertebrates. When we consider what a small proportion of biodiversity mammals actually are, it is clear how skewed this focus really is. Not only does this hinder our understanding and scientific learning, but it can also damage the perceptions and often the treatment of non-mammalian species. There is clearly a need to prioritise these taxa in future research, and to further develop our scientific understanding in order to improve the treatment and attitudes towards them.

## 7. Sentience and Advocacy

What we now know about sentience and the capacity of animals to feel pain and suffer has made a huge difference to the animal welfare movement and to how animals are treated. Unfortunately, however, there are still many industries and practices that cause immense suffering to animals, and legislation safeguarding animal welfare is still not universal. Given the overwhelming evidence of animal sentience, why is this not translated in to our treatment of animals? Do we not have enough proof, or is it just far more convenient to turn a blind eye? Considering that the majority of what we know about sentience is focused on the negative aspects, such as pain and suffering, it may be that we have simply not been using this knowledge to our best advantage. What if we were to briefly turn our attention away from the pain and suffering of animals, and instead look at the other aspects of sentience, such as the ability of animals to feel joy, form lasting friendships, hold grudges, or be empathetic? Knowledge of these remarkable commonalities between non-human animals and us may be helpful in improving people’s attitudes. If people were to see animals as the individuals that they are, with their own personalities, likes and dislikes, they may then begin to act more compassionately towards them. It is easy to compartmentalise what we know, and to temporarily forget or disassociate our activities from the impact they have. But what if by focusing on the evidence that animals are individual beings, who share many traits with us, was a way to stop that? By focusing on the positive aspects of sentience we can not only increase the humane research in the field, but we may also improve understanding, and therefore compassion and empathy towards the animals that we eat, farm, work, trade and keep. This is not to discredit the benefit of our knowledge of animal pain and suffering, but it is suggested as a complementary approach, another tool for advocates and scientists to use in their attempts to improve compassion and treatment of animals. 

Developing our understanding of animal sentience is imperative for improving animal welfare and attitudes towards animals. Concentrating on filling the gaps in our knowledge, humanely and reliably, is essential given the extent of human impact on animals. With increasing attention on animal sentience science, and the further development of humane approaches, the future of the science of animal sentience is looking ever more promising, and as a result so does the treatment of animals.
